# On the Causes Assigned as Producing Sciatica

**Published:** 1854-06

**Authors:** 


### *On the causes assigned as producing Sciatica.*

At a meeting of the Medical Society of London, Feb. 28, Mr. Hancock read a paper on Sciatica.

He alluded to the various causes assigned as producing sciatica—viz. disorder of the stomach and bowels, inflammation and disease of the sciatic nerves, syphilis, gout, obstruction and distraction of the capust coli, tumors, or accumulated fæces in the course of the nerves, effusion of fluid into the sheath of the nerve, irritation and disordered state of the kidneys, and rheumatism, either acute or sub-acute; but that, from the cases which had fallen under his observation, the prevailing opinion appeared to be that sciatica depends mostly upon rheumatism—an opinion which he considered erroneous; as, having had ample opportunities of arriving at a conclusion, he felt convinced that the cause most productive of the complaint was irritation within the pelvis, either from loaded colon or cæcum, or from tumors formed within that cavity; or, as had been suggested to him by his friend and colleague, Mr. Goldsbow, by the hæmorrhoidal vein, which, forming a complicated plexus over the sacral plexus of nerves, would, when congested and engorged, cause undue pressure and irritation of the nerve; that although he would not presume to assert, in opposition to the high authorities who differed from him, that the disease never depended upon rheumatism, still he maintained that it so seldom did so, as to constitute the exception to the general rule, and not the rule itself. Mr. Hancock then enumerated some of the modes of treatment recommended, such as bleeding, cupping, calomel and opium, colchicum, quinine, carbonate of iron, Indian hemp, acupuncture, actual cautery, blisters, moxas, puncturing the limb, and introducing morphia and creosote into punctures. He did not consider the disease difficult of cure; on the contrary, he had found it readily and speedily yield to the remedies he had adopted, which consisted in thoroughly purging the patient with small doses of croton oil, combined with blue pill, henbane, and compound extract of colocynth, and removing the sensation of bruising in the course of the nerve by the sulphate of quinine, in doses of three grains thrice daily. He had commonly given croton oil, because he had found it useful and convenient, but he did not attach any specific influence to this medicine, as he had found equally good results from turpentine and castor oil, the aloetic purgatives; and he considered that any medicine acting upon the lower intestines would be of service. He did not consider local applications at all necessary, but that they were frequently injurious, by adding to the patient's sufferings. He narrated five cases which had fallen under his care, having been previously treated for rheumatism by the remedies appropriate for the affection, but without success. In one, the sciatica had existed for nearly two years; in another, for about twelve months; and in the remainder, from four to two months. The most obstinate of these was cured in three weeks, and the remainder in a fortnight—the latter period being that usually required. He could give many more cases of a similar character, but he thought those he had described were sufficient to prove the validity of his position, that in a vast majority of instances the disease did not depend upon rheumatism, but upon the causes which he had already alluded to; and he directed the attention of the Society to an interesting fact, which he had not found noticed elsewhere—namely, that sciatica almost always occurred on the right, and very seldom indeed on the left side of the body; and when it did he believed it was caused by impaction in the sigmoid flexure of the colon. In the majority of cases the affection commenced with stiffness, weight, and pain in the lumbar region, resembling lumbago. He was inclined to attach some importance to this fact, as affording a means of diagnosis between those cases depending upon loaded intestine and those resulting from tumor in the pelvis; and that where this symptom is absent, it is desirable to institute a careful examination, not only above Poupart's ligament, but in the perineum within the tuber ischii, and also by the introduction of the finger within the rectum. He had in two instances detected the existence of tumor in the pelvis by adopting these measures; and he thought this the more desirable, from the importance of avoiding irritation as much as possible in cases of this description.

Dr. Crisp asked if the sciatic nerve was affected in all the cases referred to, since the author had spoken of the disease as an affection of the leg. On receiving an affirmative answer, he expressed an opinion that, on many grounds, the disease could not arise from pressure upon the nerve. He characterized it as one of the most excruciating of all affections, and yet is oftentimes induced by a breath of air or a mental emotion. It is also induced from malarious influences, as attested by all writers, and especially by those who had practised in malarious districts; and perhaps even the author's instance in which two brothers were affected was confirmative of this statement. He thought, on anatomical grounds, that the theory could not be sustained, since the disease was not met with in those cases in which pelvic tumors and alvine obstructions were known to exist. In reference to the influence of sex, he affirmed, in opposition to the opinion of Dr. Copland, that the disease is more frequent in males; and accounted for it on the ground that they are more exposed to changes of temperature. He then referred to the remedies which had been recommended, and approved of the rapid and repeated application of the actual cautery; of heated air, as adopted by Dr. Downing, and of the endermic application of hydrochlorate of morphia to a blistered and denuded surface.

Dr. Daniell considered that the author had been illogical in assuming that, because his treatment had been beneficial in a few cases, it would relieve in all, thus inferring the universal from the particular. If the author limited himself to such forms of sciatica as are accompanied by lumbago, he (Dr. Daniell) was of opinion that the whole profession could testify to the value of purgatives in a majority of such cases. If, however, the author spoke of sciatica in general, he could affirm, with all other authorities, that it arises from many and varied causes, and calls for different plans of treatment. If a patient be subject to rheumatism, or gout, or syphilis, he thought it fair to infer that these affections would give rise to or modify the attack of sciatica. In reference to treatment, he remarked that many patients were too enfeebled to bear the action of purgatives, and would be more relieved by a tonic plan of treatment.

Mr. Clark stated that he had recently attended a robust patient in the employ of a clothier, and had treated him ineffectually by all the ordinary modes. At length, however, on the suggestion of his assistant, he had adopted Mr. Hancock's plan of treatment, and had cured his patient in three weeks. He had given four doses of croton oil, and had obtained copious evacuations. The disease affected the right leg.

Dr. Camps considered that the error into which the author had fallen—that of inferring generals from particulars—was very common in the profession. He further was of opinion that the author had begged the question when he inferred the existence of pressure from fæcal accumulation, simply from purgatives having brought away fæcal matter. Relief obtained from the action of purgatives is equally observed in rheumatism, and a variety of other diseases, in which there is no assumption of pressure from accumulation. He approved of the exhibition of purgatives, and especially of colchicum, but considered that the treatment must be varied, according to the cause o the disease. He reminded Dr. Crisp that although sciatica is a mos painful affection, we cannot say that it is the most painful, since we have no means of measuring degrees of pain.

Dr. Richardson opposed the author's views, by stating that simple pressure upon a nerve does not give pain. Moreover, the pains in sciatica are commonly intermittent, and the remedies proposed by the author—viz., croton oil as well as turpentine, have equally good effects upon tic douloureux, and other affections of distant parts, as o the face.

Dr. Lankester was entirely at issue with the author, both in principle and practice. He regarded sciatica as a nervous affection, anc considered, therefore, that the principle of treatment should be tha applicable to the treatment of diseases of the whole nervous system This the author had overlooked. He considered that the author ha not proved his position that sciatica depends upon the pressure o fæcal matter upon the nerve; for the passage of fæcal matter afte the exhibition of a purgative is no evidence that there was fæcal accumulation, much less that the pressure caused by it had induced th pain. He had also omitted to show that the disease exists in connexion with known pelvic and intestinal tumors and fæcal accumulations. He further objected to the assumption of generals from particulars, as made by the author, and believed that purgatives would be equally beneficial in the treatment of any neuralgia. This benefit he would attribute, not only to their direct action, but to the indirect one following the action of all purgatives and depressants—viz., that of increasing the effect produced by quinine, opium, and other powerful remedies. The author had also used the quinine after the purgatives, and the cure was probably due to that remedy. In reference to the author's selection of croton oil, he reminded the fellows that Dr. Allnut had recently written a work to prove the good effects of that purgative in all cases of neuralgia, and had shown that it had given relief where all other purgatives had failed. Dr. Allnut, however, was not prepared to state whether it relieves simply by its purgative action or by some general effect upon the system. He then showed that while colchicum and croton oil, and every other remedy, may fail to effect a cure, the patient will subsequently get well without any remedies whatever. He believed the disease to arise, not from one cause only, but from many causes; and stated that in the rheumatic diathesis there is a manifest tendency to sciatica and other neuralgic manifestations.

Mr. B. Childs explained that the author had probably intended to attribute the sciatica, not to pressure, but the irritation set up by the presence of scybalæ, and stated that hip-pains in cases of hip-disease and lumber abscess were commonly relieved by injections into the rectum in the treatment of patients at the Margate Infirmary.

Mr. Gay objected to the author's deductions, but commended the practical character of the paper. The comparative infrequency of sciatica in relation to pelvic tumors and distention of the bowels, was opposed to the author's theory. Moreover, Morgagni had shown that pressure on a nerve alone will not give rise to pain. A cyst in the sheath gives no pain, so long as pressure only is exerted, but when irritation arises, pain is produced. He, therefore, thought that irritation of the nerve must exist with or without pressure, whether the cause of the sciatica be rheumatism or obstruction of the bowels. In addition to the existence of the cause of the irritation, the nerve itself must be in a condition to be irritated. He had found great benefit from the use of cupping-glasses, with or without scarification.

Dr. Webster, with the previous speakers, differed from the author in opinion as to the cause of the disease, but approved of the practical remarks contained in the paper. He approved of the plan of treatment whereby purgatives, as colchicum, are first given, and then tonics administered, and the more so in malarious cases. He cautioned the fellows as to the employment of the endermic plan of treatment, by giving the particulars of a case in which the patient had symptoms of poisoning, following the application of belladonna to the denuded surface. He believed the right leg to be more frequently affected than the left, and men than women.

Dr. O'Conner expressed his disappointment at the paper, since the author had not limited his treatment to those cases of sciatica which accompany accumulation of fæces, but had affirmed it to be applicable to that disease in general. He also showed, that even so far, the author had been anticipated by Sir Charles Bell and Dr. Allnut. He then expressed his conviction that the disease arises from very varied general causes—as rheumatism, gout, dyspepsia, and syphilis, and will not then yield to purgatives. He commended quinine and opium, on the authority of Drs. Corrigan and Graves, and Mr. Morris; and affirmed iodide of potassium and colchicum to have relieve when purgatives and other remedies had failed. He had frequently used the hot button heated under a red heat, and passed rapidly along the course of the nerve with great advantage; and also the hot air, and hot water douche of Dr. Graves.

Mr. Childs had often used the button, heated to redness, without any benefit.

The President referred to Dr. Graves' personal experience of sciatica, and reminded the fellows that that distinguished physician had been cured of sciatica by oidide of potassium and sarsaparilla, after the failure of all other remedies had determined him to withdraw from the practice of the profession. He also remarked that he (Dr. Winslow) had been a martyr to sciatica on various occasions.—He had taken it from sleeping in a damp bed at Birmingham, when on his way to hold a consultation with Sir C. Hastings, at Droitwitch, and had tried every remedy without benefit, until he had invalided himself for three weeks at Bath, when he was restored to health by the use of galvanism in the Bath waters. In reference to the influence of pressure upon a nerve as a cause of pain, he stated that when the nerve is encysted, the acupuncturation of it has given immediate relief. M. Velpeau had cured sciatica by applying the actual cautery to the external ear.

Dr. Daniell stated that the treatment to which the President had attributed his cure was that ordinarily adopted at Bath.

Mr. Potts related the particulars of a case in which the daily application of galvanism had cured, after the author's and every other mode of treatment had failed. This he considered to be a case of pure neuralgia. The patient was a man, and had the right leg affected.

Mr. Hogg had employed the author's plan with great success in several cases; and related the particulars of one case of sciatica with lumbago, in which, after having tried every remedy ineffectually for four months, Mr. Hancock had cured it by the administration of three doses of the croton oil. In a case of sciatica complicated with ascites, he had found relief from the exhibition of elaterium.

Mr. Hancock, in reply, stated that he had not overlooked the fact of irritation in connexion with pressure being a cause of sciatica, and believed it to be excited by the peristaltic action of the loaded bowels. He did not deny the existence of other causes of the disease, but considered them to constitute the exceptions.—*Dublin Medical Press, from Med. Times.*

